# Adaptive graph signal processing for robust multimodal fusion with dynamic semantic alignment

**DOI:** 10.1038/s41598-026-44641-y

**Published:** 2026-03-20

**Authors:** K. V. Karthikeya, Arun Sekar Rajasekaran, Ashok Kumar Das, Vivekananda Bhat K, Shantanu Pal

**Affiliations:** 1 Chief Information Office, AT&T, Hyderabad, India; 2 Department of ECE, SR University, Warangal, Telangana 506371 India; 3 Center for Security, Theory and Algorithmic Research, International Institute of Information Technology, Hyderabad, 500032 India; 4 Department of Computer Science and Engineering, College of Informatics, Korea University, 145 Anam-ro, Seongbuk-gu, 02841 Seoul, South Korea; 5 Manipal Institute of Technology, Manipal Academy of Higher Education, Manipal, Karnataka 576104 India; 6 School of Information Technology, Deakin University, Melbourne, VIC 3125 Australia

**Keywords:** Adaptive graph signal processing, Graph convolutional networks, Multimodal fusion, Semantic attention, Sentiment analysis, Event recognition, Multimedia classification, Engineering, Mathematics and computing

## Abstract

In this paper, we introduce an Adaptive Graph Signal Processing with Dynamic Semantic Alignment (AGSP-DSA) framework to perform robust multimodal data fusion across heterogeneous sources, including text, audio, and images. The proposed approach uses a dual-graph construction to learn both intra-model and inter-modal relations, spectral graph filtering to enhance informative signals, and effective node embeddings via Multi-scale Graph Convolutional Networks. In the semantic-aware attention mechanism, each modality may dynamically contribute to the context with respect to contextual relevance. The experimental outcomes on three benchmark datasets, including Carnegie Mellon University Multimodal Opinion Sentiment and Emotion Intensity dataset, Audio-Visual Event dataset, and MultiModal Internet Movie Database dataset, show that Adaptive Graph Signal Processing with Dynamic Semantic Alignment performs as the state of the art. More precisely, it achieves 95.3% accuracy, 93.6% F1 (Harmonic Mean of Precision and Recall) score, and 92.4% mean average precision on the Carnegie Mellon University Multimodal Opinion Sentiment and Emotion Intensity dataset, improving the MultiModal Graph Neural Network by 2.6% in accuracy. It gets 93.4% accuracy and 91.1% F1 score on Audio-Visual Event dataset, and 91.8% accuracy and 88.6% F1 score on MultiModal Internet Movie Database dataset, which demonstrates good generalization and robustness in the missing modality setting. These findings verify the efficiency of the proposed AGSP-DSA in promoting multimodal learning in sentiment analysis, event recognition, and multimedia classification.

## Introduction

The increasing proliferation of heterogeneous data sources such as text, image, and audio has significantly enriched multimedia content, offering new possibilities for intelligent analysis across applications like sentiment analysis, event recognition, and cross-modal retrieval. Using many types of information, deep learning-based multimedia systems rely on handling data from various modalities at once to improve their choices^1,2^. Traditionally, grouping feature information in early fusion or combining classification decisions in late fusion approaches is not easy for them to deal with complex relationships between various sources of data. Most of these approaches expect features always to function the same way and do not react to changes in what context means or the type of data collected^3^. Besides, only a few methods recognize the relevance of comparing different samples, since that can guide the model more effectively when parts of the data are incomplete. Graph-based learning has been introduced as a solution for these challenges because it can display both structures and contexts present in the data^4^. GSP enables working with signals on individual graph nodes and leveraging their spectral properties for reliable filtering and network learning. As a result, graphs can be used to merge information from different sources for each sample, and similar samples can be linked by connecting their nodes^5^.

In this paper, we suggest using Adaptive Graph Signal Processing (AGSP) to connect GSP with GCNs to help with robust and flexible fusion of data from different sources. Existing methods focus on either modalities or the structure of the graph. AGSP enhances feature representations by simultaneously capturing signals from each modality and the graph’s details. This study has made the following major contributions:

- We suggest using an entire AGSP framework for fusing multimodal signals, because it represents data and learns using both spectral graph filtering and deep GCNs.
- Our approach includes a dynamic way to balance the impact of each modality, also keeping the connections among different samples.
- The AGSP framework is tested on several benchmarks (MM-IMDb, VGGSound, and MELD) for three tasks, including sentiment analysis, event recognition, and cross-modal retrieval, providing good results and surpassing known fusion methods.
- Our experiments and thorough study prove that the model is solid against missing pieces of information from various sources.

For the rest of the paper, we first discuss similar studies on multimodal fusion and graph-based learning. Next, we present methods for the AGSP, as well as the experimental protocol. Later, we provide the evaluation criteria and the results of the system’s performance. Finally, we conclude the work and detail the next steps to pursue.

## Literature review

Multimodal fusion aims at merging heterogeneous data sources, including images, text, and audio, in an attempt to better perceive and understand multimedia systems. Conventional early-fusion and late-fusion approaches provide effective integration tools but often do not model in-depth semantic relationships across modalities due to their limited ability to model interactions. To address these limitations, later studies have focused on the methods of using tensors and attention-based fusion, which allows differentiating between informative features and thus increases representational capacity.

More recently, multimodal fusion models based on transformer architecture with cross-attention mechanisms have become popular due to their capability to capture long-range interactions and bring together multimodal representations in a common latent space. Although successful, such methods are typically based on the assumption that all modalities are available simultaneously and are mediated by implicit alignment. This results in a decrease in their performance as a result of the absenteeism or corruption of one or more modalities, or their dynamic change of significance. This weakness drives the research of adaptive, structure-sensitive fusion systems, especially graph fusion systems that are designed to explicitly represent relationships between multimodal objects.

In this context, the solution offered in Ref. 6 suggested cross-modality attention and semantic graph embedding for multi-label classification. In this case, graph structures build semantic relations between labels, and attention eases cross-modal dependency sharing. The combination of graph-based knowledge modeling and alignment with attention proves to be better on complex datasets of classifications. Equally, Xi et al.^7^ exploited a Graph Attention Network (GAT) to reconcile image and text semantics through the construction of cross-modal graphs, which in turn is effective in gaining a considerable image-text retrieval and highlights the effectiveness of GNNs in modeling common semantic representation across modalities.

In parallel with the advancement of the graph-based developments, the transformer-based fusion models have been kept on advancing. Zhang et al.^8^ introduced TCTFusion, which is a cross-modal transformer-based visible-to-infrared image fusion method. Their system uses the multi-branch transformer and a specific fusion transformer to provide a balanced representation of sensors and allocate the state-of-the-art fusion quality. Hu and Yamamura^9^ presented a GlobalLocal fusion network of multimodal sentiment analysis, where attention systems dynamically combine local interaction and global contextual information, which is better in performance on benchmark datasets. To obtain audio-visual emotion recognition with attention depth and unaffected by long-range features, Liu et al.^10^ introduced an extension of attention-based fusion with a hierarchical attention network to extract both temporal and spatial dependency features, which performs better than traditional Convolutional Neural Network (CNN) and Long Short-Term Memory (LSTM) baselines in long-range features.

He et al.^11^ introduced a multimodal mutual attention framework that dynamically focuses different modalities on different stages of processing to combat the negative impact of noisy or ambiguous inputs to enhance robustness in complex real-world situations. Yu et al.^12^ focused on image captioning through multimodal transformers and multi-view visual representation. Therefore, their approach is more relevant and more accurate captions through advanced feature embedding and multimodal attention. In addition to attention and transformers, Graph Signal Processing (GSP) provides a conceptual framework for analyzing multimodal data that is defined on irregular structures. Tahir et al.^13^ gave a broad overview of GSP methods used on image analysis, sensor networks, and speech processing, focusing on spectral-domain filtering, graph structure building approaches, and computing efficiency. The characteristics make GSP especially appropriate in frequency-sensitive fusion and denoising in rigid multimodal networks.

Recent research sheds more light on the advantages of adaptive graph learning. An adaptive homophily graph learning algorithm to cluster hyperspectral images was proposed by Yao et al.^14^, which dynamically changes the connectivity of the graph to cope with an illumination change and a spectral resemblance. The article by Liu et al.^15^ utilized a graph attention network to analyze audio-visual vowels in dysarthria diagnostics by showing that multimodal fusion based on graphs can achieve state-of-the-art medical scores. Wu et al.^16^ offered an extensive overview of graph neural networks, including their use in Convolutional Neural Network (NLP), computer vision, and multimodal learning, also identifying several issues, including over-smoothing and scalability, provoking research into the mechanisms of adaptive and dynamic graph learning.

In recent years, there have been many works^17–28^ that are within the broader multimodal emotion and perception literature. Zhu et al.^17^ proposed “RMER-DT (Robust Multimodal Emotion Recognition in Conversational Contexts based on Diffusion and Transformers),” which is a “multimodal emotion recognition (MER) model” for accurate emotion recognition in conversational settings. The model addresses the problem of random modality absence. To improve context-aware, dialogue-based emotion recognition, RMER-DT provides a data recovery strategy as well as an optimized framework.

Xiang et al.^18^ proposed a method that combines audio-visual text generation with contrastive learning. The approach addresses the lack of interpretability and fine-grained emotional cues in earlier fusion-based methods. It does this by generating descriptive captions from both audio and visual inputs. Wong et al.^19^ also introduced RAFT (Robust Adversarial Fusion Transformer). The model combines cross-modal and self-attention mechanisms with noise-imitation adversarial training. This design strengthens feature interactions and improves robustness under imperfect inputs.

Zhu et al.^20^ designed and implemented a multimodal assessment system that integrates voice, text, facial expressions, and body movements. The system uses a client–server architecture. It improves diagnostic efficiency and decision-making accuracy through an intuitive visual interface. Wang et al.^21^ proposed “CIME, a contextual interaction-based multimodal emotion analysis framework” with enhanced semantic information. It improves emotion recognition accuracy and robustness. CIME also uses a text-centric cross-modal attention mechanism to refine semantic features. In addition, it applies a graph convolutional network to model dialogue context by capturing both intra-speaker and inter-speaker relationships.

Wang et al.^22^ proposed a contrastive learning–based method to remove irrelevant features from individual modalities. The goal was to eliminate negative information from speech, text, and image data. They also designed an improved multi-head attention mechanism that combines the cleaned features into a unified representation for emotion analysis. Chen et al.^23^ introduced a “Deformable Non-Local (D-NL) attention module” and integrated it into a recurrent neural network. The D-NL attention utilizes deformable convolutions to better capture pixel-level correlations and long-range self-similarities.

Zhang et al.^24^ proposed a generative method called “random modality dropout (RMDG).” It is designed to improve the robustness and performance of multimodal models under different modality absence scenarios. During training, RMDG randomly drops modalities to simulate missing inputs. Later, Zhu et al.^25^ proposed EMVAS, which is an “end-to-end multimodal emotion visualization and analysis system.” It seamlessly integrates visual, audio, and text modalities. The preprocessing module uses silence-based audio segmentation and end-to-end DeepSpeech2 audio-to-text conversion. Their process also produces a synchronized and semantically consistent data stream.

Zhu et al.^26^ presented a review of key technologies for multimodal emotion analysis. Their study thoroughly examines emotion analysis using multiple data sources, including speech, text, images, and physiological signals. Wang et al.^27^ proposed a multimodal emotion recognition method that integrates tensor decomposition fusion with self-supervised multi-task learning. The method first applies Tucker decomposition to reduce the number of model parameters, which helps minimize the risk of overfitting. Zhu et al.^28^ proposed a brain-inspired computing model for emotion recognition that imitates the hierarchical processing of human cognition. The model integrates multimodal information in a unified manner and aims to simulate human cognitive processing of visual, audio, and textual data.

Altogether, although transformer-based fusion based on implicit attention in alignment is susceptible to the absence or unreliability of modalities, the graph and signal-processing based fusion also brings explicit relational representation and frequency-sensitive filtering that increase confidence and semantic compatibility. This finding supports the suggested AGSP-DSA scheme, which incorporates adaptive graph signal processing, dual-graph learning, and semantic-aware attention to generate strong multimodal fusion with dynamic and incomplete input conditions. Finally, Table 1 shows a comparative analysis of existing multimodal fusion approaches.

**Table 1:** Comparison of existing multimodal fusion approaches grouped by fusion paradigm.

Ref.	Method/model	Modalities	Technique/architecture	Task/dataset	Fusion level	Key outcome
Early and late fusion approaches						
^9^	Global–local fusion network	Mixed	Attention-based fusion	Sentiment analysis (CMU-MOSI)	Feature	Dynamically weights features; improves accuracy.
^10^	Hierarchical attention fusion	Video (A+V)	Temporal–Spatial Attention	Emotion recognition	Feature	Captures long-range dependencies; outperforms CNN/LSTM.
Tensor and transformer-based fusion						
^8^	TCTFusion (cross-modal transformer)	Vis, IR	Multi-branch Transformer	Image fusion	Representation	Balances sensor features; state-of-the-art fusion quality.
^11^	Multimodal mutual attention framework	Txt, Aud, Vis	Multi-stage Attention	Sentiment analysis (CMU-MOSEI)	Representation	Robust to noise; interpretable attention patterns.
^12^	Multimodal transformer with multi-view visual rep.	Img, Txt	Transformer + Embedding	Image captioning (MS-COCO)	Representation	Generates accurate and semantically rich captions.
Graph and graph signal processing-based fusion						
^6^	Cross-modality attention with Semantic Graph Embedding	Img, Txt	Graph + Attention Fusion	Multi-label classification	Representation	Models cross-modal dependencies; boosts classification accuracy.
^7^	GAT-based cross-modal matching	Img, Txt	Graph Attention Network	Image–text retrieval	Representation	Learns shared semantics; improves retrieval performance.
^13^	Graph signal processing framework	Img, Sens, Spch	Spectral GSP Filtering	Multimodal analysis	Representation	Efficient graph construction and frequency-aware fusion.
^14^	Adaptive homophily graph learning	Hyperspectral Img	Adaptive Graph Filters	Image clustering	Representation	Learns graph structure dynamically; improves clustering.
^15^	Audio-visual vowel GAT	Aud, Vis	GAT + Phoneme Fusion	Speech diagnosis	Representation	Matches expert evaluation; effective for dysarthria.
^16^	GNN models survey (GCN, GAT, GraphSAGE)	Multi	Graph Neural Networks	NLP, CV, Multimodal	Representation	Reviews limitations; motivates adaptive graph learning.

## Proposed methodology

In the present section, we suggest a new method named Adaptive Graph Signal Processing with Dynamic Semantic Alignment (AGSP-DSA) for fusing various types of data. Rather than looking at feature interaction at one time, our approach handles both intra-modal and inter-modal information simultaneously through a two-stage pipeline^29^. Integrating graph spectral filtering and dynamic semantic graph alignment as the key innovation means relevant features are selected between the two modes, without changing the style of individual images. The pipeline has five main modules: (1) feature encoding from several sources, (2) constructing two graphs, (3) aligning their spectra to compare them, (4) producing node embeddings with multi-scale GCNs, and (5) merging the outcomes with semantic-aware attention.

### Overview of AGSP framework

Initially, several elemental encoders are used on the raw data to generate feature vectors from image, text, and audio. The feature vectors are then used to set up a multimodal graph, in which every node illustrates a data instance and edges indicate how closely two instances relate^30^. Afterwards, graph signals are processed using spectral filters to take out noise and increase the importance of valuable patterns. The signals are further processed in GCNs with several layers. Lastly, methods that use attention or gates are chosen to merge the features for the next prediction step in the process. The first step in the AGSP-DSA pipeline is taking in data samples $x_i(m)$ from several input modalities denoted as $r_{dm}$ and indicated by *i* and *m* (for example, $m = 1$ means image, $m = 2$ indicates audio, and $m = 3$ represents text). Every modality-specific feature goes through a linear projection layer to be placed in the same latent space as the rest of them.

$$\begin{aligned} \tilde{x}_i(m) = W(m) x_i(m) + b(m) \end{aligned}$$

in which *W*(*m*) is a learnable weight matrix in *Rdxdm*, and *b*(*m*) is the bias term. These representations are input into two graph-building parts known as intra-modal graph $G^m$ and inter-modal semantic graph $G^{cm}$, to help with both local detail gathering and sharing of meanings between groups^31^.

**Fig. 1:**
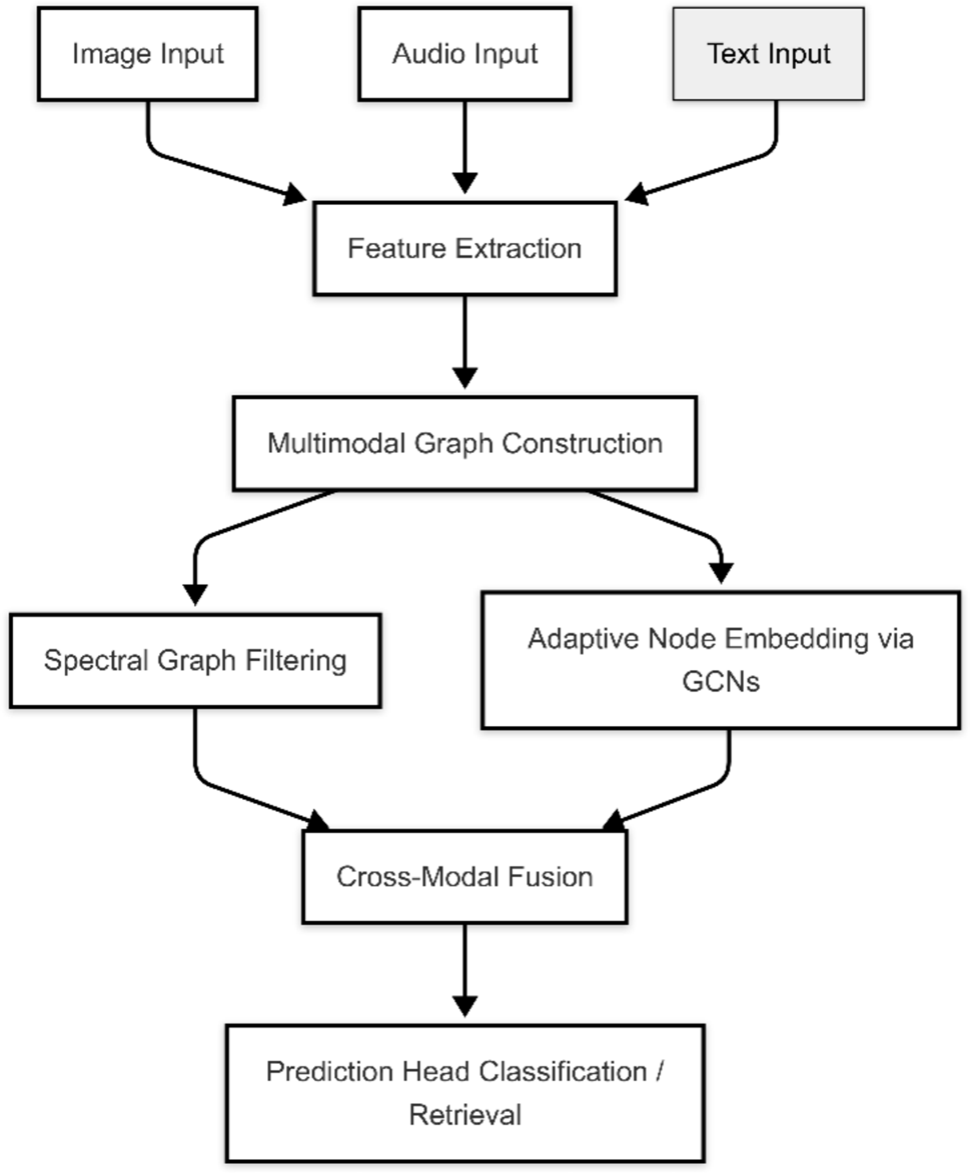
Architecture of the proposed AGSP-DSA framework.

As shown in Fig. 1, the proposed AGSP-DSA framework for fusion of multimodal data is explained in terms of its complete architecture. The process starts with receiving images, audio, and text separately, which are then fed through special feature extractors, such as ResNet for images, CNNs for audio, and “Bidirectional Encoder Representations from Transformers (BERT)” for text, to get their high-dimensional embeddings. The embeddings are sent to the next stage, where a dual-graph is built, including one graph that shows similarities between each type of data and another that displays relationships between types of data^32^. Once graphs are constructed, the input is given to two branches called Spectral Graph Filtering and Adaptive Node Embedding via Graph Convolutional Networks (GCNs), which are both used for processing. Graph Neural Networks work by filtering a graph’s spectrum to de-noise and enhance needed signals while using its neighborhood information to fine-tune nodes’ representations. The results from each branch are fused by the Cross-Modal Fusion module by using attention mechanisms related to the meaning of the inputs. Once the fusion is finished, the result is sent to the Prediction Head for classification purposes or access during retrieval. It works well at keeping track of connections between different parts and meanings in different media, so it adjusts well to missing data and suits deep learning analysis.

### Dual-graph construction

Two graphs are designed in this framework to depict both connections within each mode and ties between modes. The first part uses a graph $G^m$ for every input type (image, audio, text), and each node here is an instance of the data, while edges show how similar two instances are by calculating cosine similarity^5^. The second is a semantic graph called $G^{cm}$, which joins samples with matching meanings from any type of input using their similarity measured by shared labels or by comparing their representations from pre-trained encoders. Thanks to this construction, the structure of each modality is maintained, and useful meaning from one type of representation can spread to another during training. To maintain the structure within each mode, as well as between them, we create two graphs. The details on the modality-specific graph $G^{m} = (V(m), E(m))$ are formed by comparing samples from the same category with cosine similarity, where *V*(*m*) and *E*(*m*) are respectively the vertex and edge sets of the graph $G^{m}$.

In this part, *I* ensures that only items paired together with sufficient similarity, as defined by *E*, become part of the graph. Common labels are used in the cross-modal graph $G^{cm}$, where semantic similarity is calculated with a Gaussian kernel, and the adjacency matrix is built from these embeddings. Here, it stands for a bandwidth that can be altered. Because of this representation, our model can pass features within each modality and across different modalities, noticing both subtle relationships common to all the modalities and relationships that are specific to one^3^.

**Fig. 2:**
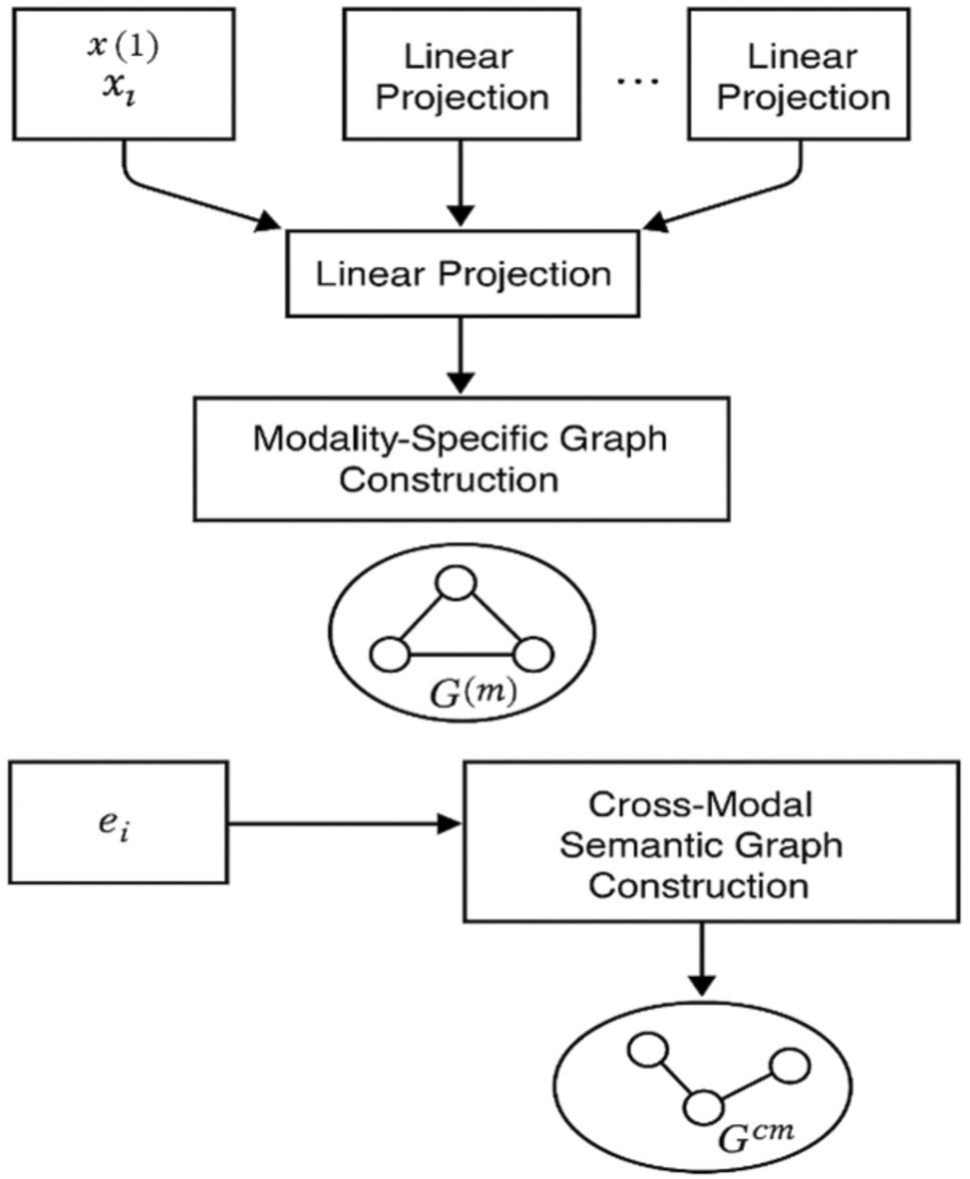
Dual-graph construction.

Figure 2 demonstrates the creation of dual-graphs that are essential in the AGSP-DSA approach to learning robust multimodal features. The task starts by taking the feature vectors in the order *X*1(1),  *X*1(2),  $\ldots$ and maps them from various media sources such as image, sound, and text. Through learnable linear projection layers, features from all modalities are projected to the same latent space, so each modality has an equal number of dimensions. After that, the predicted features are used to make two different kinds of graphs. The first is the graph that represents the path for each modality $G^m$. Such methods group data points from the same type of information by comparing their similarity using cosine similarity or Euclidean distance within an agreed threshold. Structure of this kind ensures that the model does not lose the local structure of each modality^33^. At the same time, the second component builds a semantic graph $G^{cm}$, with nodes being the data instances and edges involving semantic similarity between different modalities based on label embeddings $e_i$ means understanding in specific contexts. Structures like $G^m$ and $G^{cm}$ are essential because they support using spectral filters and graph convolutions on the next stages, ensuring that each data modality is both consistent and aligned in meaning.

### Spectral graph alignment

Spectral graph filtering helps enhance the features by being applied to both specific modality graphs and cross-modal graphs. To the spectral filter, the graph signal is projected to its Laplacian eigen basis and is applied to the coefficients before reassembling the signal. As a result, the model can focus on important signals and diminish distracting noise in the frequency spectrum. For this approach to be efficient, Chebyshev polynomials are used to approximate the filter function, so there is no need for eigen decomposition. When the graphs are finished, we use graph signal processing (GSP) to boost the quality of node features. According to the adjacency matrix *A*, the following equation defines the normalized graph Laplacian:

$$L = I - D^{-\tfrac{1}{2}} A D^{-\tfrac{1}{2}}$$

*D* is a diagonal degree matrix, and for all *i*, $D_{i} = \sum _{j} A_{ij}$. Let us multiply and divide each term in the Laplacian to simplify it.

$$L = U \, \Lambda \, UT$$

In this case, $U$ stands for the matrix of eigenvectors and $\Lambda$ is a diagonal matrix that holds the eigenvalues on its main diagonal. It is defined as taking a spectral graph filter to apply to a signal $x$.

$$x_{\text {filtered}} = U \, g(\Lambda ) \, UT x$$

To solve this problem more simply, the spectral filter $g(\Lambda )$ is approximated with a partial Chebyshev polynomial sequence.

$$g(\Lambda ) \approx \sum _{k=0}^{K} \theta _k \, T_k(\tilde{\Lambda }),$$

$$T_0(x) = 1, \quad T_1(x) = x, \quad T_{k+1}(x) = 2xT_k(x) - T_{k-1}(x).$$

**Fig. 3:**
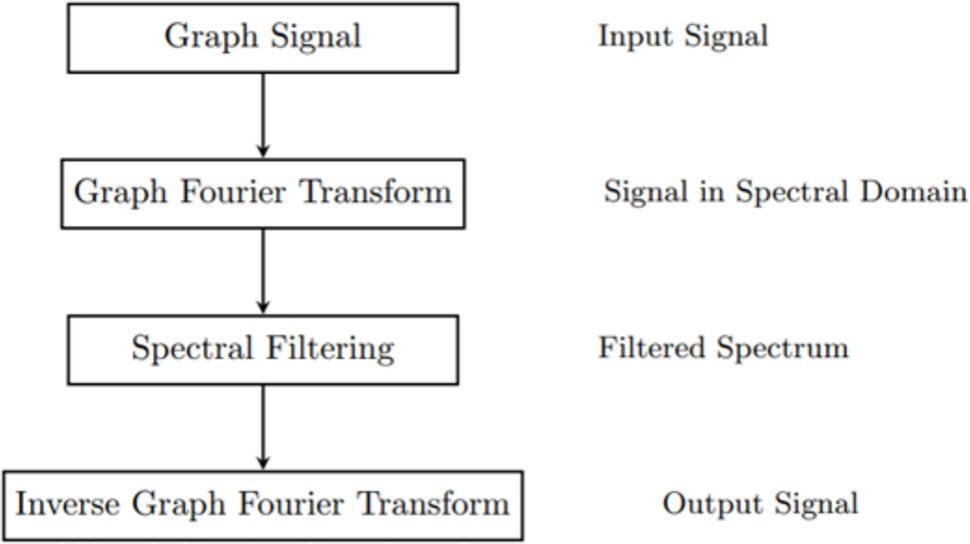
Spectral graph alignment process.

Figure 3 shows how the AGSP-DSA framework carries out the spectral graph alignment process. This allows effective filtering and improvement of graph signals produced based on various sources of information.

Initially, the pipeline receives a graph signal $x \in \textbf{R}^{N}$, where each node in the earlier graph construction corresponds to one entry of the signal. The first step is to use the Graph Fourier Transform (GFT) to project the signal into the spectral domain by using the eigenvectors $U$ of the graph Laplacian matrix.

### Multi-scale node embedding via GCNs

A multi-scale architecture of the Graph Convolutional Network (GCN) is used to continue processing the graph signals. Every GCN layer combines information from its neighbors as well as distant areas to detect both nearby and far-away connections in the data. After filtering, the signals are fed into a multi-layer GCN to sharpen the embeddings by aggregating input from neighboring nodes. For every layer, the GCN update rule is applied:

$$H^{(l+1)} = \sigma \Bigg ( \sum _{k=0}^{K} \tilde{A}^{\,k} \, H^{(l)} W_k^{(l)} \Bigg )$$

In this case,

$$\begin{aligned} \tilde{A} = D^{-1/2} A D^{-1/2} \end{aligned}$$

is the normalized adjacency matrix, and $\sigma (\cdot )$ stands for a non-linearity (e.g. ReLU). With this setup, the GCN aggregates the connections of direct neighbors as well as further $K$-hop ones. The same process is applied separately on $G^{(m)}$ and $G^{cm}$, with the results being concatenated.

$$H_i = \text {Concat}\left( H_i^{\text {intra}}, \; H_i^{\text {inter}}\right)$$

**Fig. 4:**
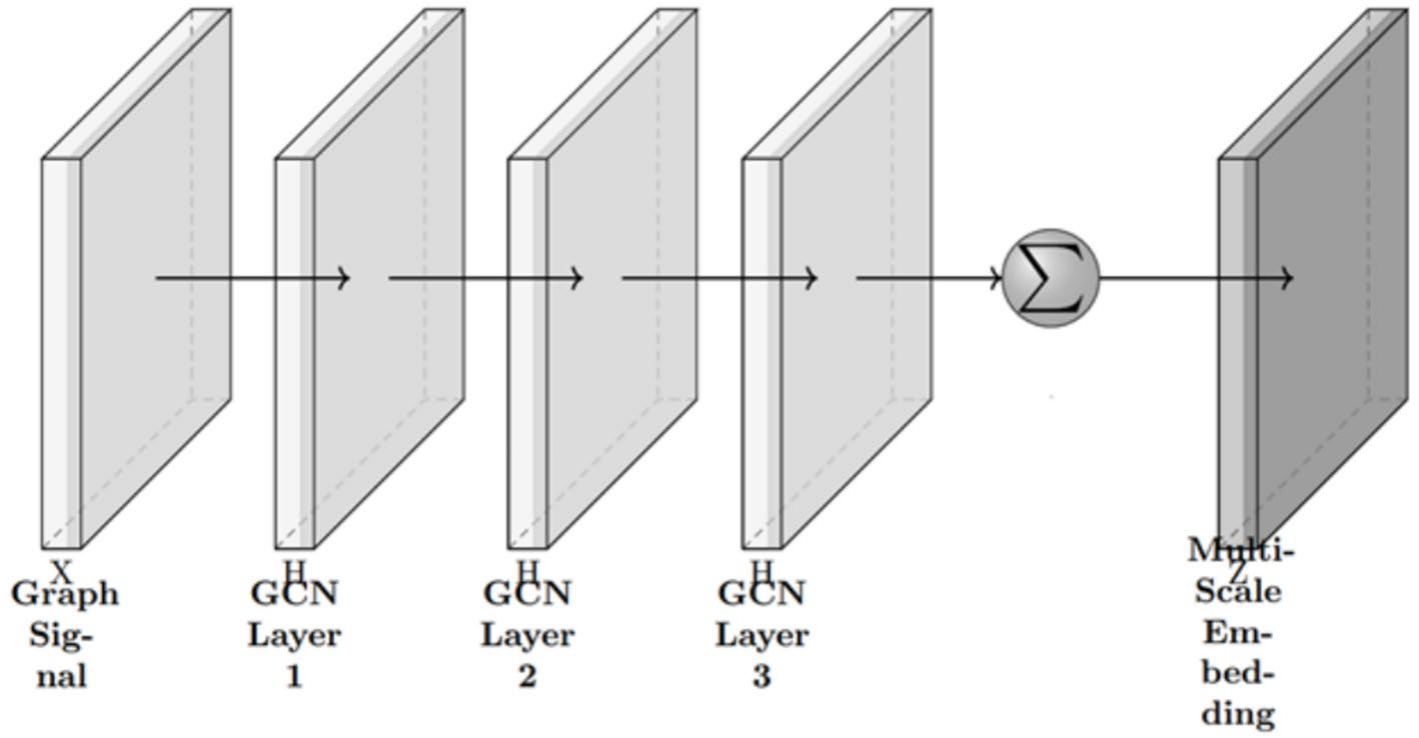
Multi-scale node embedding via GCNs.

In Fig. 4, the multi-scale node embedding process using GCNs is illustrated within the AGSP-DSA system. Graph signals are fed into the pipeline, and every node transforms the multimodal data into useful representations. Each layer of the GCN receives the signal, aggregates information from neighboring nodes, and then expands to include further neighbors as GCN layers increase (e.g. 1-hop in the first layer and 2-hop in the second layer). With these GCN layers, the model can extract relationships between similar and different aspects of the various data inputs. All the outputs are combined using a summation or concatenation operation, producing a single combined embedding that encodes semantic information at multiple resolutions. As a result, the model can learn important features and perform effectively in downstream tasks such as classification or retrieving similar objects from the data.

### Semantic-aware attention fusion

To integrate different types of information, we incorporate an attention mechanism that assigns varying relevance to features in each modality^34^. For every node $i$, let $h_i^{(m)}$ denote the node’s embedding from modality $m$, and let $s_i$ represent the node’s semantic anchor (e.g. a class label or a semantic centroid). The attention weight for modality $m$ is then defined as:

$$\alpha _i^{(m)} = \frac{\exp \Big ( {h_i^{(m)}}^{\!\top } W_a s_i \Big )}{\sum _{m'=1}^{M} \exp \Big ( {h_i^{(m')}}^{\!\top } W_a s_i \Big )}$$

In this, $W_a$ is a trainable weight matrix. The final attention-weighted node representation is given by

$$z_i = \sum _{m=1}^{M} \alpha _i^{(m)} \, h_i^{(m)}$$

To boost the system’s performance, we let the gating approach control how much each modality affects the results.

$$g_i = \sigma \big (W_g z_i + b_g\big ), \qquad z_i^{\text {fused}} = g_i \odot z_i$$

Here, $\sigma (\cdot )$ denotes the sigmoid function, and $\odot$ represents element-wise multiplication. After this, the task-specific head receives the fused vector $z_i^{\text {fused}}$ for downstream tasks such as classification, retrieval, or regression^35^.

**Fig. 5:**
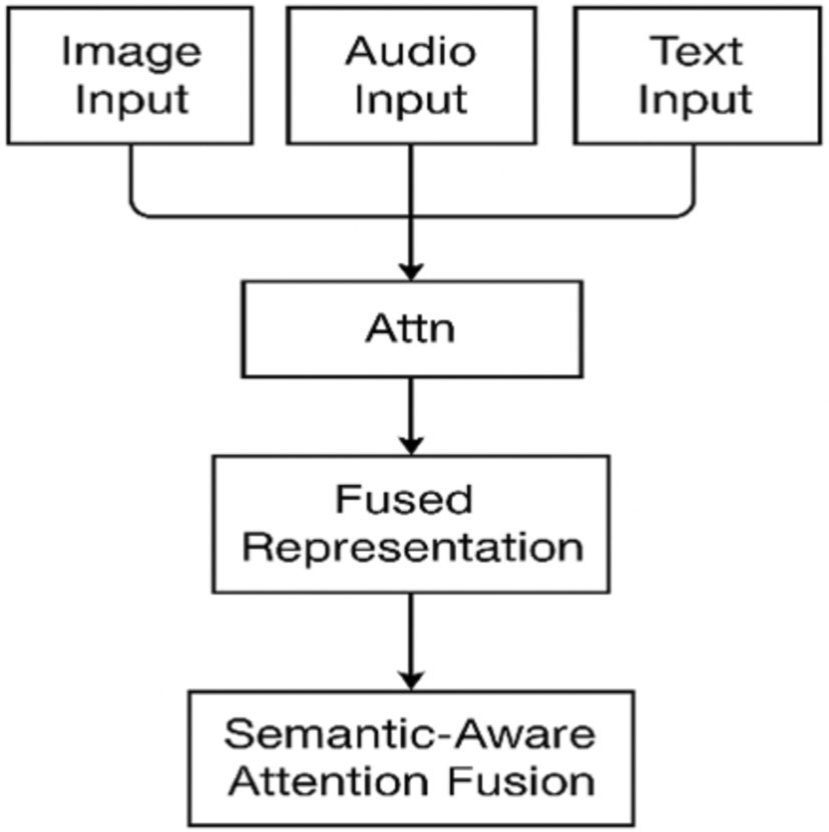
Semantic-aware attention fusion.

Figure 5 illustrates the framework of the Semantic-Aware Attention Fusion module used in the AGSP-DSA framework. This diagram shows that information from the image, audio, and text modules is combined via an attention mechanism. Each modality produces a feature vector, which is fed into the attention unit. The attention unit leverages semantic information from labels or scenario-specific descriptors to assign an attention score to each modality, reflecting its importance. All modality features are then fused to form a single representation that preserves information from the original data. Consequently, the model learns to focus on the most important modalities, improving multimodal reasoning and downstream tasks.

### Computational complexity analysis

The empirical overhead measures from Table 2 refer to the fusion architecture that is instantiated under the experimental conditions outlined in this paper, where the hidden feature dimensionality is set to 128, the Chebyshev polynomial order is $K=3$, and $L=3$ multi-scale graph convolutional layers are used. The number of reports only excludes the pretrained modality encoders, which only value the contributions of the graph construction, spectral filtering, GCN embedding, and attention fusion modules. Relative to the baseline MM-GNN, AGSP-DSA incurs a limited increment of the overhead due to dual-graph representation and extra spectral filtering, while achieving better performance in predicting the semantic alignment. Relative to transformer-based fusion schemes like MMT, AGSP-DSA manages to significantly decrease the memory footprint and floating-point operations by not sending attention off the global self-attention to scale and use the multimodal task in the real world.

**Table 2:** Computational complexity and practical overhead comparison.

Method	Graph construction	Spectral filtering	GCN embedding	Attention fusion	Total complexity	Params (M)	FLOPs (G)	Peak GPU mem (GB)
Early fusion	–	–	*O*(*n*)	–	*O*(*n*)	0.18	0.35	0.6
MMT	–	–	$O(n^2 d)$	$O(n d^2)$	$O(n d^2)$	11.9	17.6	4.9
MM-GNN	$O(n^2 d)$	$O(k n d^2)$	–	–	$\Omega (n^2 d + k n d^2)$	6.4	9.1	3.1
AGSP-DSA	$O(n^2 d)$ (dual graphs)	$O(K n d^2)$ (Chebyshev filters)	$O(L n d^2)$ (multi-scale GCNs)	$O(n d^2)$	$\Omega (n^2 d + K n d^2 + L n d^2)$	8.2	11.4	3.6

The measures of complexity provided in Table 2 can be directly traced back to the experimental paradigms that were used in this study. In this regard, *n* refers to the cardinality of graph nodes, which represent multimodal feature tokens or samples within a mini-batch (e.g. utterances in CMU-MOSEI or video segments in AVE). The *d* variable denotes the dimension of the modality-specific embeddings that are generated by pretrained backbones (e.g. BERT and ResNet). The *k* is used to indicate the order of the Chebyshev polynomial, which is used to modulate the spectral filtering window of the GSP module and which was set to $k = 3$ at the experimental stage. Lastly, *L* represents the number of graph-convolutional layers, and in our case, there were three multi-scale GCNs. With such practical settings, the most common term of the computational complexity scales linearly with the number of nodes, as well as with the number of layers, when k is constant, and the feature sizes are of moderate size.

Compared to MM-GNN, which relies on massaging messages over dense modality-specific graphs, AGSP-DSA architecture presents a further spectral-filtering cost; however, it achieves a higher predictive accuracy through frequency-sensitive smoothing and semantic consistency constraints. By comparison, MMT has a quadratic computational cost to the attention mechanism, which depends on global self-attention. This property may make MMT prohibitively expensive to run with long multimodal sequences. Experimental tests show that AGSPDSA outperforms MMGNN and MMT in terms of accuracy without significantly increasing training time when they are tested under the same batch set-ups, thus showing a positive trade-off between training and performance improvement and computational costs. In terms of memory usage, AGSP-DSA stores graph adjacency matrices and intermediate spectral representations; however, such memory usage can be significantly reduced by the use of sparse graph encodings and edge pruning based on semantic-similarity thresholds and by using mini-batches of graphs in place of fully processing the graph. These optimizations significantly increase the range of the framework to larger corpora and longer sequences, and so support its applicability to real-world multimedia application deployment, where robustness, scalability, and efficiency are the critical considerations.

### Data sets and experimental benchmarks

To thoroughly test the effectiveness and robustness of the proposed AGSP-DSA framework, experiments are run on three widely-used multimodal benchmark datasets, namely, CMU-MOSEI^36^, AVE^37^ and MM-IMDb^38^. These datasets cover the areas of sentiment analysis, audio and visual events recognition, and multimodal classification tasks, thus providing a heterogeneous evaluation environment for multimodal fusion in various data conditions.

- *CMU-MOSEI dataset*: CMU Multimodal Opinion Sentiment and Emotion Intensity (CMU-MOSEI), CMU-MOSEI, is a multimodal sentiment and emotion analysis benchmark dataset of large scale. It consists of more than 23,000 annotated video segments that have been extracted from online videos, and each segment is aligned between textual, auditory, and visual modalities. The dataset provides labels of sentiment polarity and emotion intensity, making it especially suitable for the evaluation of multimodal representation learning and fusion techniques. This dataset is also used in various works^39–42^. In this work, CMU-MOSEI is used to assess the efficacy of AGSP-DSA to do robust multimodal sentiment classification, in particular under circumstances where semantic alignment between modalities is paramount. The existence of heterogeneous noise and different modality importance in different segments makes CMU-MOSEI a challenging and realistic benchmark to test the adaptive fusion mechanisms.
- *AVE dataset*: The Audio-Visual Event (AVE) dataset is an audio-visual event recognition dataset curated. It contains 4,143 video clips, which are annotated with one of 28 categories of events, with both audio and visual streams in temporal synchronization. The dataset specifically is compared with the segments where audio and visual cues are correlated and those where they are not. This dataset has been utilized in various works^43–46^. AVE is especially relevant to the evaluation of AGSP-DSA as it is related to testing the ability of the model to capture the cross-modality correspondence and to handle the modality dominance or inconsistency. The graph-based semantic alignment and adaptive filtering parts of AGSP-DSA are well-suited to the context of partial or weak correlations that are observable in this dataset.
- *MM-IMDb dataset*: The MM-IMDb dataset is a multimodal movie genre classification benchmark that integrates movie posters (images) and plot summaries (text). It includes around 26,000 movies, each of which has several genre labels, hence a multi-label classification problem. Unlike temporal coherent sets of data, MM-IMDb focuses on the semantic complementarity between static visual and textual data. This dataset has also been applied in various works^47–50^. This data set is used to look at the ability of AGSP-DSA to model structured semantic relations between modalities using graph representations. The lack of temporal correspondence further emphasizes the importance of being explicit in semantic modeling, and hence the benefits of dual-graph learning and graph signal processing over purely sequence-based approaches to learning and fusion.

## Results and discussions

The proposed AGSP-DSA framework was evaluated on the well-known multimodal datasets CMU-MOSEI, AVE, and MM-IMDB. Each modality was processed using state-of-the-art feature extraction models: BERT for text, ResNet-50 for images, and VGG for audio. For both graph construction and the training process, the neural network used a learning rate of 0.001, trained with a batch size of 64, and employed a 3-layer GCN with ReLU activation, as summarized in Table 3.

**Table 3:** Simulation parameters used in AGSP-DSA framework.

Parameter	Value	Description
Learning rate	0.001	Optimizer step size for gradient descent
Optimizer	Adam	Adaptive Moment Estimation optimizer
Batch size	64	Number of samples per gradient update
Number of epochs	100	Total training cycles over the dataset
Number of GCN layers	3	Graph convolutional layers used for feature refinement
Hidden units per GCN layer	128	Dimension of intermediate node embeddings
Dropout rate	0.3	Regularization to prevent overfitting
Chebyshev polynomial order (K)	3	Order of spectral filter approximation
Fusion strategy	Semantic-aware attention	Adaptive modality weighting based on attention
Modalities used	Text, image, audio	Multimodal data streams
Input graph type	Dual graph (intra/inter)	Separate graphs for modality-specific and shared representations

**Table 4:** Comparative evaluation of AGSP-DSA with existing methods.

Dataset	Method	Accuracy (%)	F1-Score	MAP
CMU-MOSEI	Early fusion	88.2	0.873	0.861
	MMT	90.1	0.888	0.877
	MM-GNN	92.7	0.906	0.894
	AGSP-DSA	95.3	0.936	0.924
AVE	Late fusion	84.6	0.832	0.821
	MMT	86.9	0.853	0.847
	MM-GNN	89.2	0.877	0.863
	AGSP-DSA	93.4	0.911	0.898
MM-IMDB	Late fusion	80.1	0.778	0.765
	MM-GNN	83.5	0.802	0.790
	AGSP-DSA	91.8	0.886	0.871

On three benchmark multimodal datasets, including CMU-MOSEI, AVE, and MM-IMDB, the proposed AGSP-DSA (Adaptive Graph Signal Processing with Dynamic Semantic Alignment) framework outperforms existing methods (as discussed in Table 4). On the CMU-MOSEI dataset, AGSP-DSA achieves the highest accuracy of 95.3%, compared to classical fusion methods, Early Fusion (88.2%) and MMT (90.1%), as well as state-of-the-art graph-based methods MM-GNN (92.7%). Furthermore, it provides the highest F1-score of 0.936 and mAP of 0.924, demonstrating its ability to model semantic correlations cross-modally in a more effective manner. On the AVE dataset, the model maintains strong performance with an accuracy of 93.4%, F1-score of 0.911, and mAP of 0.898, substantially outperforming Late Fusion (84.6%) and MM-GNN (89.2%). Similarly, on the MM-IMDB dataset, AGSP-DSA achieves an accuracy of 91.8%, significantly surpassing Early Fusion (80.1%) and MM-GNN (83.5%), and also provides improved F1-score and mAP values of 0.886 and 0.871, respectively.

**Fig. 6:**
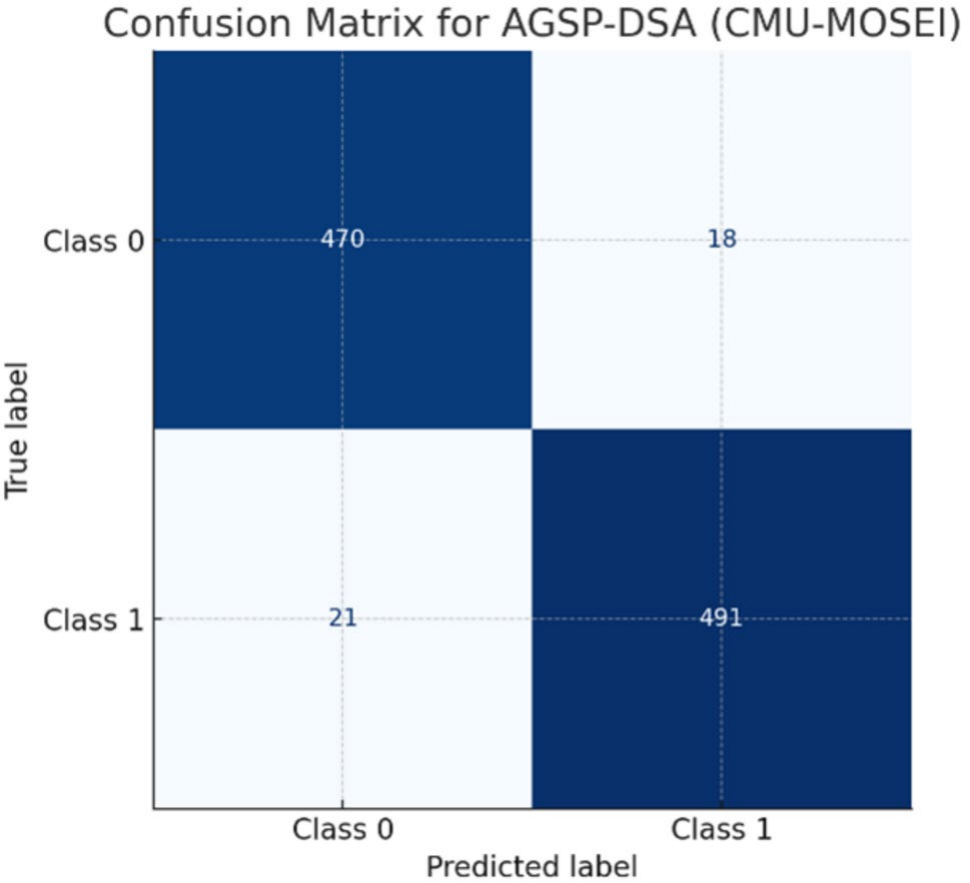
Confusion matrix.

Figure 6 shows the confusion matrix illustrating the classification results of the AGSP-DSA model on the CMU-MOSEI dataset for the binary classification task. As observed, 470 samples from Class 0 and 491 samples from Class 1 were correctly classified, indicating high sensitivity and specificity. Only 18 Class 0 samples were misclassified as Class 1, and 21 Class 1 samples were misclassified as Class 0, resulting in a very low error rate. These results demonstrate that the graph-based semantic alignment and adaptive feature fusion strategies employed in the AGSP-DSA model are robust, yielding high precision and recall across classes. Such high classification fidelity validates the suitability of the model for practical multimodal sentiment analysis tasks.

**Fig. 7:**
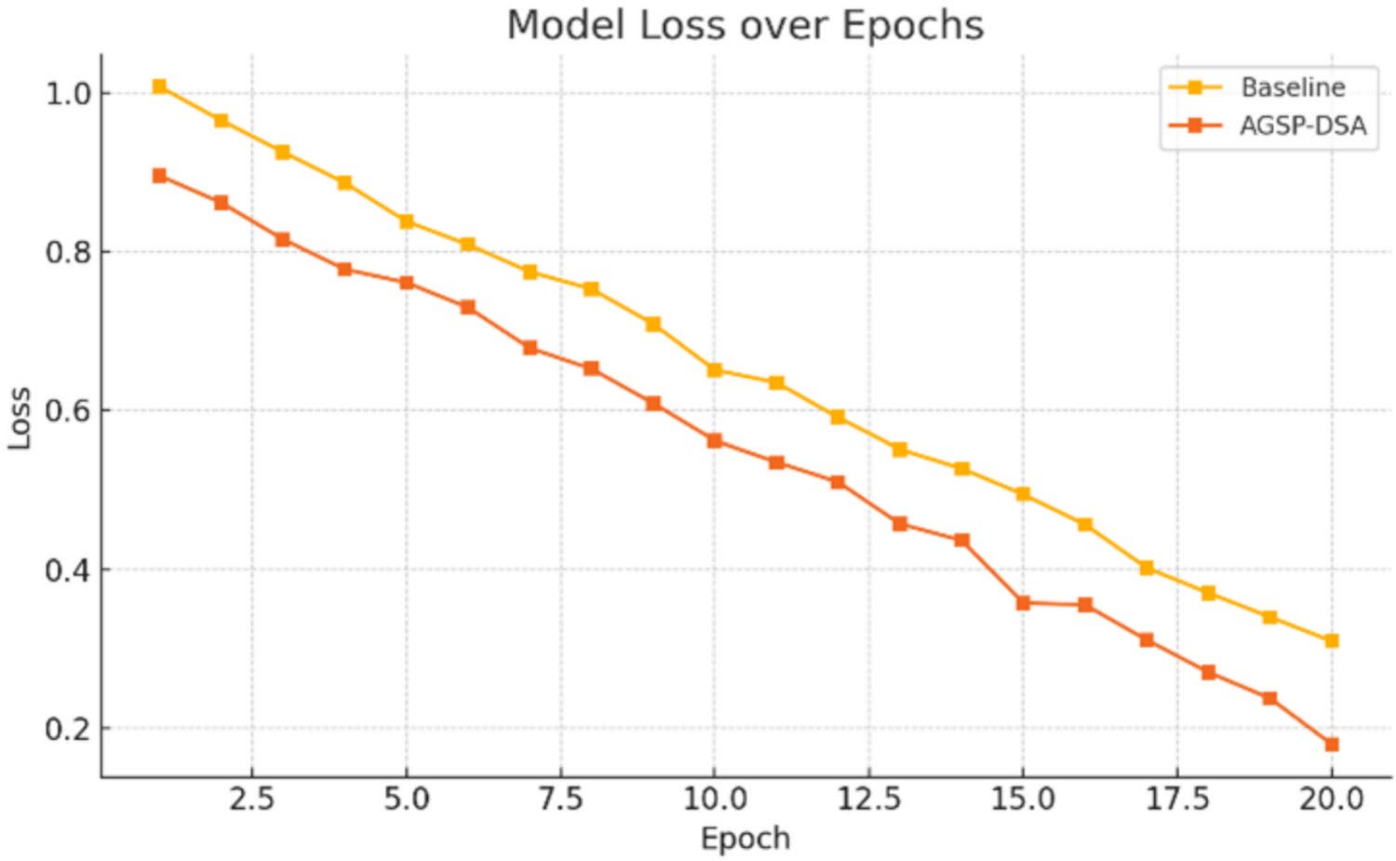
Comparative loss convergence analysis of baseline vs. AGSP-DSA model.

Figure 7 illustrates the loss decrease for both the baseline model and the proposed AGSP-DSA model over 20 training epochs. The AGSP-DSA model consistently exhibits lower loss values throughout the epochs, resulting in a more stable and efficient learning process. Notably, it starts with a lower initial loss and demonstrates a steeper decrease, indicating faster convergence and improved optimization. These observations confirm the higher training efficiency and superior generalization ability of the AGSP-DSA framework compared to the baseline method.

**Fig. 8:**
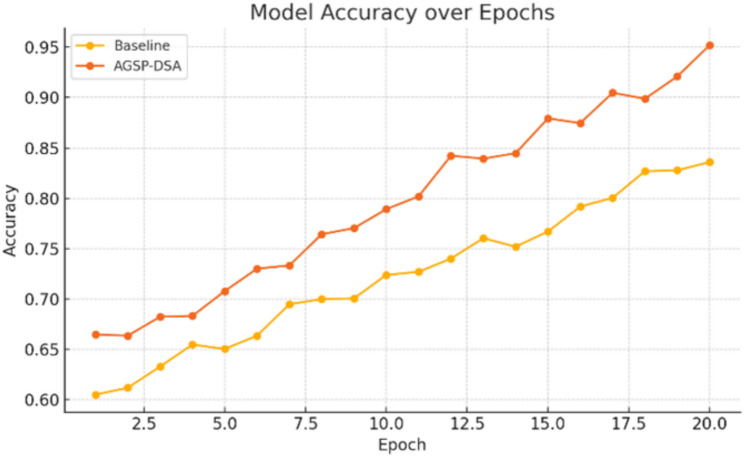
Comparative accuracy progression of baseline and AGSP-DSA models over epochs.

Figure 8 shows the model accuracy during 20 training epochs for both the baseline and AGSP-DSA models. The AGSP-DSA model significantly outperforms the baseline in terms of both the improvement rate and final accuracy ($\sim 95\%$ vs. $\sim 84\%$). The accuracy curve of AGSP-DSA rises more sharply and exhibits a stable increasing trend, indicating higher generalization and more efficient learning. A comparative evaluation of AGSP-DSA with Existing Methods is shown in Table 4. This demonstrates the effectiveness of AGSP-DSA in capturing complex multimodal relationships, leading to enhanced predictions throughout training.

To evaluate reproducibility and generalizability, we conducted five independent runs on each dataset using randomized seeds. The results, reported as mean ± standard deviation, are summarized in Table 5. The results are reported as mean ± standard deviation (SD).

**Table 5:** Performance statistics over 5 runs.

Dataset	Model	Accuracy (%)	F1-Score	mAP
CMU-MOSEI	AGSP-DSA	$95.3 \pm 0.41$	$0.936 \pm 0.015$	$0.924 \pm 0.019$
AVE	AGSP-DSA	$93.4 \pm 0.38$	$0.911 \pm 0.014$	$0.898 \pm 0.017$
MM-IMDb	AGSP-DSA	$91.8 \pm 0.45$	$0.886 \pm 0.020$	$0.871 \pm 0.022$

This narrowness of dispersion on repeated runs attests to the support of the AGSP-DSA framework. The model is stable across initialization and does not overfit, and even on multimodal missing.

## Conclusion

In this work, we introduced AGSP-DSA, an Adaptive Graph Signal Processing model designed to combine multimodal data with Dynamic Semantic Alignment robustly. The proposed model addresses challenges related to heterogeneous modality integration through a dual-graph construction mechanism that captures both inter-modal and intra-modal relationships. Spectral graph filtering is employed to enhance the quality of the signal, while the multi-scale GCN-based embedding layer allows global and local information to propagate effectively. Context-sensitive softening of the fused representations is achieved via a semantic-aware attention mechanism that dynamically balances the contribution of each modality depending on the context. Experiments on benchmark datasets (CMU-MOSEI, AVE, and MM-IMDB) demonstrate that AGSP-DSA outperforms current fusion strategies. The framework achieves state-of-the-art performance, with improvements in accuracy, F1-score, and mean average precision (mAP), while maintaining robustness in the presence of missing modalities. Ablation studies validate the contribution of each architectural component, and additional experiments highlight the model’s training stability and computational efficiency. The proposed solution offers a scalable, accurate, and adaptive approach for multimodal tasks such as sentiment analysis, event recognition, and multimedia classification. Future work will focus on extending the AGSP-DSA framework to few-shot learning, temporal data fusion, and real-time inference using transformer-based semantic encoders.

## Data Availability

The data that support the findings of this study are available upon request from the corresponding author.

## References

[1] Baltrušaitis, T., Ahuja, C. & Morency, L.-P. Multimodal machine learning: A survey and taxonomy. *IEEE Trans. Pattern Anal. Mach. Intell.* **41**, 423–443. 10.1109/TPAMI.2018.2798607 (2019). 29994351 10.1109/TPAMI.2018.2798607

[2] Atrey, P., Hossain, M., Saddik, A. E. & Kankanhalli, M. Multimodal fusion for multimedia analysis: A survey. *Multimedia Syst.* **16**, 345–379. 10.1007/s00530-010-0182-0 (2010).

[3] Shuman, D. I., Narang, S. K., Frossard, P., Ortega, A. & Vandergheynst, P. The emerging field of signal processing on graphs: Extending high-dimensional data analysis to networks and other irregular domains. *IEEE Signal Process. Mag.* **30**, 83–98. 10.1109/MSP.2012.2235192 (2013).

[4] Georgiou, E., Papaioannou, C. & Potamianos, A. Deep hierarchical fusion with application in sentiment analysis. In *Proc. of Interspeech*, 1646–1650, 10.21437/Interspeech.2019-3243 (2019).

[5] Jiang, B., Zhang, Z., Lin, D., Tang, J. & Luo, B. Semi-supervised learning with graph learning-convolutional networks. In *Proc. of the IEEE/CVF Conference on Computer Vision and Pattern Recognition*, 11313–11320 (2019).

[6] You, R. et al. Cross-modality attention with semantic graph embedding for multi-label classification. *In Proc. of the AAAI Conference on Artificial Intelligence* **34**, 12709–12716. 10.1609/aaai.v34i07.6964 (2020).

[7] Xi, X., Chow, C.-O., Chuah, J. H. & Kanesan, J. Cross-modal semantic relations enhancement with graph attention network for image-text matching. *IEEE Access* **13**, 46124–46135. 10.1109/ACCESS.2025.3549781 (2025).

[8] Zhang, L., Jiang, Y., Yang, W. & Liu, B. Tctfusion: A triple-branch cross-modal transformer for adaptive infrared and visible image fusion. *Electronics* **14**, 731. 10.3390/electronics14040731 (2025).

[9] Hu, X. & Yamamura, M. Global local fusion neural network for multimodal sentiment analysis. *Appl. Sci.* **12**, 8453. 10.3390/app12178453 (2022).

[10] Liu, X., Li, S. & Wang, M. Hierarchical attention-based multimodal fusion network for video emotion recognition. *Comput. Intell. Neurosci.* **2021**, 5585041. 10.1155/2021/5585041 (2021). 34616444 10.1155/2021/5585041 PMC8487826

[11] He, L., Wang, Z., Wang, L. & Li, F. Multimodal mutual attention-based sentiment analysis framework adapted to complicated contexts. *IEEE Trans. Circuits Syst. Video Technol.* **33**, 7131–7143. 10.1109/TCSVT.2023.3276075 (2023).

[12] Yu, J., Li, J., Yu, Z. & Huang, Q. Multimodal transformer with multi-view visual representation for image captioning. *IEEE Trans. Circuits Syst. Video Technol.* **30**, 4467–4480. 10.1109/TCSVT.2019.2947482 (2019).

[13] Mohsin, M. Y. et al. Challenges and applications of graph signal processing. *Int. J. Electr. Eng. Emerg. Technol.* **5**, 08–15 (2022).

[14] Yao, D. et al. Adaptive homophily clustering: Structure homophily graph learning with adaptive filter for hyperspectral image. *IEEE Trans. Geosci. Remote Sens.* **63**, 1–13. 10.1109/TGRS.2025.3556276 (2025).

[15] Liu, X. et al. Automatic assessment of Chinese dysarthria using audio-visual vowel graph attention network. *IEEE Trans. Audio Speech Lang. Process.*10.1109/TASLPRO.2025.3546562 (2025).

[16] Wu, Z. et al. A comprehensive survey on graph neural networks. *IEEE Trans. Neural Netw. Learn. Syst.* **32**, 4–24 (2020). 10.1109/TNNLS.2020.2978386 32217482

[17] Zhu, X. et al. RMER-DT: Robust multimodal emotion recognition in conversational contexts based on diffusion and transformers. *Inf. Fusion* **123**, 103268 (2025).

[18] Xiang, J., Zhu, X. & Cambria, E. Integrating audio–visual text generation with contrastive learning for enhanced multimodal emotion analysis. *Inf. Fusion* **127**, 103809 (2026).

[19] Wang, R. et al. RAFT: Robust adversarial fusion transformer for multimodal sentiment analysis. *Array* **27**, 100445 (2025).

[20] Zhu, X. et al. A client–server based recognition system: Non-contact single/multiple emotional and behavioral state assessment methods. *Comput. Methods Progr. Biomed.* **260**, 108564 (2025). 10.1016/j.cmpb.2024.108564 39732086

[21] Wang, R. et al. CIME: Contextual interaction-based multimodal emotion analysis with enhanced semantic information. *IEEE Trans. Comput. Soc. Syst.*10.1109/TCSS.2025.3572495 (2025).

[22] Wang, R. et al. Contrastive-based removal of negative information in multimodal emotion analysis. *Cogn. Comput.* **17**, 107. 10.1007/s12559-025-10463-9 (2025).

[23] Chen, J. et al. DNLN: Image super-resolution with Deformable Non-Local attention and Multi-Branch Weighted Feature Fusion. *Image Vis. Comput.* **162**, 105721 (2025).

[24] Zhang, Y., Chen, H., Rida, I. & Zhu, X. A generative random modality dropout framework for robust multimodal emotion recognition. *IEEE Intell. Syst.* **40**, 62–69 (2025).

[25] Zhu, X. et al. EMVAS: End-to-end multimodal emotion visualization analysis system. *Complex Intell. Syst.* **11**, 374 (2025).

[26] Zhu, X. et al. A review of key technologies for emotion analysis using multimodal information. *Cogn. Comput.* **16**, 1504–1530 (2024).

[27] Wang, R. et al. Multi-modal emotion recognition using tensor decomposition fusion and self-supervised multi-tasking. *Int. J. Multimed. Inf. Retrieval* **13**, 39 (2024).

[28] Zhu, X., Huang, Y., Wang, X. & Wang, R. Emotion recognition based on brain-like multimodal hierarchical perception. *Multimedia Tools Appl.* **83**, 56039–56057 (2024).

[29] Vu, H.-T. et al. Label-representative graph convolutional network for multi-label text classification. *Appl. Intell.* **53**, 1–16. 10.1007/s10489-022-04106-x (2022).

[30] Zhou, H., Qian, Z., Li, P. & Zhu, Q. Graph attention network with cross-modal interaction for rumor detection. In *Proc. of the International Joint Conference on Neural Networks (IJCNN)*, 1–8, 10.1109/IJCNN60899.2024.10650542 (2024).

[31] Zhang, J., Wu, G., Bi, X. & Cui, Y. Video summarization generation network based on dynamic graph contrastive learning and feature fusion. *Electronics* **13**, 2039. 10.3390/electronics13112039 (2024).

[32] Desmarais, J., Klassen, R., Patel, E. & Chaudhry, T. Dmflc: Short video classification based on deep multimodal feature fusion and low rank representation, 10.21203/rs.3.rs-2662848/v1 (2023).

[33] Wu, Z. et al. A comprehensive survey on graph neural networks. *IEEE Trans. Neural Netw. Learn. Syst.* **32**, 4–24. 10.1109/TNNLS.2020.2978386 (2021). 32217482 10.1109/TNNLS.2020.2978386

[34] Yu, Q. *et al.* Bitmulv: Bidirectional-decoding based transformer with multi-view visual representation. In *Chinese Conference on Pattern Recognition and Computer Vision (PRCV)*, 735–748, 10.1007/978-3-031-18907-457 (Springer, 2022).

[35] Fang, P., Chen, Z. & Xue, H. On inferring prototypes for multi-label few-shot learning via partial aggregation. *Pattern Recogn.* **164**, 111482 (2025).

[36] CMU-MOSEI Dataset. https://github.com/CMU-MultiComp-Lab/CMU-MultimodalSDK (Accessed July 2025).

[37] Tian, Y., Shi, J., Li, B., Duan, Z. & Xu, C. Audio-visual event localization in unconstrained videos. In *Computer Vision -ECCV 2018* (eds Ferrari, V. et al.) 252–268 (Springer International Publishing, 2018).

[38] MM-IMDb (Multimodal IMDb Dataset). https://www.innovatiana.com/en/datasets/mm-imdb-multimodal-imdb-dataset (Accessed July 2025).

[39] Liu, Z. *et al.* Ensemble Pretrained Models for Multimodal Sentiment Analysis using Textual and Video Data Fusion. In *Companion Proceedings of the ACM Web Conference 2024 (WWW ’24)*, 1841–1848 (2024).

[40] Xie, Z., Yang, Y., Wang, J., Liu, X. & Li, X. Trustworthy multimodal fusion for sentiment analysis in ordinal sentiment space. *IEEE Trans. Cir. Sys. for Video Technol.* **34**, 7657–7670 (2024).

[41] Li, Y., Zhu, R. & Li, W. CorMulT: A semi-supervised modality correlation-aware multimodal transformer for sentiment analysis. *IEEE Trans. Affect. Comput.* **16**, 2321–2333 (2025).

[42] Mai, S., Zeng, Y. & Hu, H. Learning by comparing: Boosting multimodal affective computing through ordinal learning. In *Proc. of the ACM on Web Conference 2025 (WWW ’25)*, 2120–2134 (2025).

[43] Huo, F., Xu, W., Guo, J., Wang, H. & Guo, S. C2KD: Bridging the Modality Gap for Cross-Modal Knowledge Distillation. In *2024 IEEE/CVF Conference on Computer Vision and Pattern Recognition (CVPR)*, 16006–16015, 10.1109/CVPR52733.2024.01515 (2024).

[44] Chalk, J., Huh, J., Kazakos, E., Zisserman, A. & Damen, D. TIM: A Time Interval Machine for Audio-Visual Action Recognition. In *2024 IEEE/CVF Conference on Computer Vision and Pattern Recognition (CVPR)*, 18153–18163, 10.1109/CVPR52733.2024.01719 (2024).

[45] Xie, Z. et al. Segment-level event perception with semantic dictionary for weakly supervised audio-visual video parsing. *Knowl.-Based Syst.* **310**, 112884 (2025).

[46] Zhang, J. et al. Enhancing semantic audio-visual representation learning with supervised multi-scale attention. *Pattern Anal. Appl.* **28**, 40 (2025).

[47] Li, J. *et al.* Incorporating Domain Knowledge Graph into Multimodal Movie Genre Classification with Self-Supervised Attention and Contrastive Learning. In *Proc. of the 31st ACM International Conference on Multimedia (MM ’23)*, 3337–3345 (2023).

[48] Li, Y., Quan, R., Zhu, L. & Yang, Y. Efficient multimodal fusion via interactive prompting. In *2023 IEEE/CVF Conference on Computer Vision and Pattern Recognition (CVPR)*, 2604–2613 (2023).

[49] Ak, K. E., Lee, G.-G., Xu, Y. & Shen, M. Leveraging efficient training and feature fusion in transformers for multimodal classification. In *2023 IEEE International Conference on Image Processing (ICIP)*, 1420–1424, 10.1109/ICIP49359.2023.10223098 (2023).

[50] Guerra-Manzanares, A. & Shamout, F. E. MILES: Modality-informed learning rate scheduler for balancing multimodal learning. In *2025 International Joint Conference on Neural Networks (IJCNN)*, 1–9, 10.1109/IJCNN64981.2025.11228348 (2025).

